# Reply to the Letter of Charre et al. “Mis-Genotyping of Some Hepatitis D Virus Genotype 2 and 5 Sequences Using HDVdb”

**DOI:** 10.3390/v12111278

**Published:** 2020-11-09

**Authors:** Zainab Usman, Stoyan Velkov, Ulrike Protzer, Michael Roggendorf, Dmitrij Frishman, Hadi Karimzadeh

**Affiliations:** 1 Department of Bioinformatics, Wissenschaftszentrum Weihenstephan, Technische Universität München, 85354 Freising, Germany; zainabasrar@gmail.com (Z.U.); d.frishman@wzw.tum.de (D.F.); 2 Institute of Virology, Technische Universität München, 81675 Munich, Germany; stoyan.velkov@tum.de (S.V.); protzer@tum.de (U.P.); michael.roggendorf@tum.de (M.R.); 3 Division of Clinical Pharmacology, University Hospital, LMU Munich, 80337 Munich, Germany

We thank Charre and colleagues for spotting the mis-annotation of sequences in our database, which was caused by human error. The problem has been corrected. In the future, we recommend submitting bug reports directly to one of the e-mail addresses indicated on the HDVdb Web page.

